# A Systematic Review and Meta-Analysis of Tourniquet Pressures in Upper Limb Surgery

**DOI:** 10.3390/jcm14061938

**Published:** 2025-03-13

**Authors:** Kayen Chan, Rawan Jaibaji, Eleanor Barker, Cyrus Talwar, Calver Pang

**Affiliations:** 1 Addenbrookes Hospital, Cambridge University Hospitals, Cambridge CB2 0QC, UK; kayen.chan3@nhs.net (K.C.); rawan.jaibaji1@nhs.net (R.J.); 2 School of Clinical Medicine, University of Cambridge Medical Library, Cambridge CB2 0SP, UK; 3 Frimley Health Foundation Trust, Wrexham Park Hospital, Slough SL2 4SH, UK; c.talwar@nhs.net; 4 Department of Surgical Biotechnology, Division of Surgery and Interventional Science, Faculty of Medical Sciences, University College London, London WC1E 6BT, UK

**Keywords:** upper limb surgery, tourniquet pressures, meta-analysis, hand surgery

## Abstract

**Background:** Tourniquet pressures used in upper limb surgery are commonly standardized at 250 mmHg. These higher tourniquet pressures have been associated with an increased risk of complications, such as neural compression injury and soft tissue damage. However, there has been limited consensus as to the use of lower tourniquet pressures and their efficacy. This systematic review and meta-analysis aims to examine the current evidence for the use of lower tourniquet pressures in upper limb surgery, comparing them to the standard tourniquet pressures of 250 mmHg and, in some cases, 300 mmHg. This study is registered on PROSPERO (CRD42024511501). The primary outcome was the adequacy and quality of the bloodless field achieved at lower pressures. Secondary outcomes were assessed when reported, including the operative time, pain, and complications. **Methods:** The databases Medline (via Ovid), Embase (via Ovid), Cochrane, Web of Science (Core Collection) and Scopus, ClinicalTrials.gov, EU Clinical Trials Register, and ISRCTN registry were searched from inception to January 2025. The inclusion criteria included patients undergoing upper limb surgery using regional or general anesthetic with the use of a pneumatic tourniquet. A total of 1994 studies were identified, of which 12 met the inclusion criteria for review and 8 studies were used in the meta-analysis. Risk of bias was assessed using the ROBINS-I and RoB-2 tools. **Results:** The sample size was 1427 patients with a mean age of 46.8 years. All studies showed a bloodless surgical field at lower tourniquet occlusion pressures. The meta-analysis showed the overall mean tourniquet inflation pressure, estimated using a random effects model, to be 169.3 mmHg with a 95% confidence interval of 144.9–193.6. However, the heterogeneity in the reported results is significant (*p* < 0.0001) and is a limitation to this review. **Conclusions:** This systematic review highlights the benefit of the use of a lower tourniquet pressure (below the standard 250 mmHg) to produce an adequate surgical field and influence procedural outcomes.

## 1. Introduction

The use of the tourniquet dates to 1674 when Etienne Morel, a French Army surgeon, used a stick to twist a soldier’s bandage until the bleeding from his injury ceased [1]. In upper limb surgery, tourniquets play an important role in ensuring a bloodless surgical field, enhancing the surgeon’s visibility and improving surgical precision. By applying controlled pressure to an extremity, blood flow is occluded to the region distal to the tourniquet, enabling the surgeon to operate with reduced bleeding and without the distraction of venous congestion, potentially reducing operating times [2,3]. However, the use of tourniquet pressure requires careful management to avoid complications such as tissue ischemia, nerve damage, and postoperative pain [2,4]. The pressure should be sufficient to stop the arterial flow without causing permanent tissue damage or nerve injury.

Conventionally, a standard tourniquet pressure of 250 mmHg is used during upper limb surgery or a fixed amount of pressure above arterial systolic pressure (typically 100 mmHg above systolic pressure) [5]. Although there have been suggestions of lower inflation pressures, there is lack of consensus and standardization of the optimal minimum efficient tourniquet pressure [4]. Joint guidelines released by the British Orthopedic Association (BOA), British Society for Children’s Orthopedic Surgery (BSCHO), and the British Society for Surgery of the Hand (BSSH) recommend that for patients over the age of 16, the tourniquet pressure should be calculated using systolic BP (sBP) plus 50–100 mmHg in the upper limb [6]. In patients where their systolic blood pressure is lower than 150 mmHg, the standard tourniquet pressure of 250 mmHg may be higher than required to ensure a bloodless field, increasing their risk of developing adverse effects from the tourniquet pressure such as neural compression injuries, soft tissue damage, vasomotor palsy, and pain [2,4,7]. The use of the lowest effective tourniquet inflation pressure has been advocated to reduce the risk of these tourniquet-related injuries.

This systematic review aims to examine the current evidence for the use of lower tourniquet pressures in upper limb surgery, comparing them to the standard tourniquet pressures of 250 mmHg and, in some cases, 300 mmHg. The primary outcome was the adequacy and quality of the bloodless field achieved at lower tourniquet occlusion pressures. The limb occlusion pressure (LOP) in this review is defined as the lowest tourniquet pressure required to stop blood flow to the limb distal to the cuff. This can be determined by the presence of arterial pulse with Doppler flow or through a bloodless surgical field [8,9]. Secondary outcomes were assessed when reported, including the operative time, pain, and complications.

## 2. Materials and Methods

This systematic review was conducted in accordance with the items of the PRISMA-NMA (Preferred Reporting Items for Systematic Reviews and Meta-Analyses—Network Meta-Analyses) checklist and registered on PROSPERO (CRD42024511501).

### 2.1. Search Strategy

The databases Medline (via Ovid), Embase (via Ovid), Cochrane, Web of Science (Core Collection), Scopus, ClinicalTrials.gov, EU Clinical Trials Register, and ISRCTN registry were searched from inception to January 2025 by EB. The search strategy was peer-reviewed by two librarian colleagues of EB using the Peer Review of Electronic Search Strategies (PRESS) checklist [10] and evaluated against the PRISMA-S guidelines [11]. The completed PRISMA-S checklist is shown in the Supplementary Material. Databases were searched by EB separately rather than multiple databases being searched on the same platform. The search syntax was adapted for each database and to account for variation between thesaurus terms/controlled vocabulary across each database. Results were imported to Endnote 21 by EB for deduplication using the method outlined by Bramer et al. [12]. The full search strategies used in each database are documented in full below.

### 2.2. Study Inclusion/Exclusion Criteria

Clinical studies that investigated different tourniquet pressures in patients undergoing upper limb surgery were included. Studies were included in the review if they assessed any clinical outcomes of variable tourniquet pressures in upper limb surgery. Letters to the editor, narrative studies, in vitro, and animal or cadaveric studies were excluded. Articles in which the full text was not available in English were also excluded.

Inclusion criteria were as follows:

1. Patient population undergoing upper limb surgery under regional or general anesthesia;
2. Case series, cohort studies, or randomized controlled trials;
3. Use of upper limb tourniquet during surgery;
4. Any outcome measure reported.

Exclusion criteria were as follows:

1. Upper limb surgeries carried out under local anesthesia;
2. Articles not reporting original research, e.g., narrative review articles;
3. Articles in which full text is not available in English;
4. Conference abstracts that have not been published as full-text publications;
5. Articles that are duplicate publications, i.e., have been previously published as full-text publications;
6. Articles in which studies were conducted using data from non-human studies, or articles presenting cadaveric studies.

### 2.3. Study Selection and Data Extraction

Once the search was conducted, a first scan of the study titles was performed to identify studies with relevant titles. From these, a second reading was then carried out to screen the abstracts of these studies. Finally, a third reading allowed inclusion of the relevant studies. The eligibility criteria were assessed by two reviewers (KC and CP) independently at each stage, and any discrepancies or disagreements during the process were resolved through discussion with a third reviewer (RJ).

Data were extracted on study design, number of patients, average age of patients, type of surgery performed, mean tourniquet pressure used, follow up, and complications. The main evaluation criteria were the achievement of an adequate bloodless field, with secondary outcomes including operative times and any complications experienced by patients.

### 2.4. Statistical Analysis

We used descriptive and basic statistical methods to synthesize the results of our systematic review. Mean age of the results from individual studies are shown if provided by the author. For each study, the mean and standard deviation of the minimal tourniquet inflation pressure used to achieve a bloodless surgical field together with the sample sizes used were reported. We conducted a single arm meta-analysis as there are no control groups in most of the studies considered. In our primary meta-analysis, we pooled mean tourniquet inflation pressures from each study using a random effects model, a more conservative approach that allows for between-study variability. Whether data from studies can be appropriately pooled can be established by examining the degree of heterogeneity (that is, the extent to which outcomes vary across studies). We tested heterogeneity by using both the Q statistic (Chi-squared statistic) and the $I^{2}$ statistic; a significant Q statistic (*p*-value < 0.05) indicated significant heterogeneity (that is, a significant difference in mean tourniquet inflation pressures across studies). In addition, we also conducted a sensitivity analysis of the data by comparing a fixed effects model against the random effects model to pool the effect sizes. To investigate the heterogeneity between the studies, we carried out two sub-group analyses based on the age and BMI of the participants. In both subgroup analyses, mean tourniquet inflation pressures for the subgroups were again pooled using random effects models. All results were visualized using forest plots. The level of significance for all statistical tests was α=0.05. All analyses were conducted in R (version 4.3.2).

### 2.5. Quality Assessment

Appraisal of the study quality of the included studies was carried out independently by 3 reviewers (KC, CP, and RJ). Criteria developed by the Centre for Evidence Based Medicine was used to assess the overall quality of evidence across study designs. The risk of bias was evaluated using the Risk of Bias In non-randomized Studies of Interventions (ROBINS-I) (as shown in Table 1) as well as the Revised Cochrane risk of bias tool for randomized trials (RoB-2) (as shown in Table 2). Levels of risk of bias include “high”, “moderate”, “low”, and “serious”. Each published article was assessed based on five aspects (patient selection, ascertainment, outcome, follow-up, and reporting) [13,14].

## 3. Results

The study selection process and the results of the literature search are presented in the Appendix A. The search identified 1994 articles, and 12 studies were included in this systematic review.

### 3.1. Study Characteristics

The sample size of the 12 included articles was 1427 patients with a mean participant age of 46.8 years. Upper limb surgeries include carpal tunnel decompression, ganglion excision, tendon repairs, trigger finger release, and open reduction internal fixation of fractures. Follow up ranged from immediately post-operatively to 1 week post-operatively. Four included studies were randomized controlled trials (RCTs) [4,5,20,21], and the remaining studies were non-randomized studies, including five retrospective comparative studies, three prospective cohort studies, and one prospective case series [2,3,7,15,16,17,18,19].

### 3.2. Tourniquet Pressures

Tourniquet pressures were calculated in various ways across the studies. All studies investigated tourniquet pressure below the standard of 250 mmHg. These ranged from 90 mmHg to 275 mmHg. Three studies had a control group, with two having the standard of 250 mmHg [3,20] and one with 300 mmHg [15].

One study (5) measured arterial occlusion pressure (AOP) (using Doppler arterial flow) +20 mmHg, with the average maximal tourniquet pressure calculated at 186.91 ± 12.9 with an average AOP of 161.85 ± 15.75. Van Roekel et al. [15] started the operation at 300 mmHg and then gradually reduced pressure during the operation until capillary bleeding occurred; it was recommended that in a normotensive patient, a pneumatic tourniquet pressure of 200 mmHg would suffice as in all cases, it was found that the tourniquet pressure in which capillary bleeding occurred was at least 40 mmHg below the calculated tourniquet pressure. This was a similar technique used by Othman et al. [16]. Liu et al. [21] used an equation which included AOP estimates. Levy et al. [19] used a Doppler opening pressure measurement and subsequently used the systolic BP (sBP) of the patient. Moore et al. [17] used Doppler blood flow occlusion measurements. Kasem et al. [5] used AOP and LOP estimates in order to determine the lowest tourniquet inflation pressure. Azad et al. [2] used pre-determined guidelines based on a patient’s systolic blood pressure (60 mmHg was added to the pressure for an sBP of less than 130 mmHg, 80 mmHg for an sBP between 131 and 190 mmHg, and 100 mmHg for an sBP > 191 mmHg). Table 3 is a summary of the tourniquet pressures used and the methods.

### 3.3. Adequacy of Surgical Field

The adequacy of the surgical field was either assessed through surgeon ranking or through monitoring of bleeding during surgery. Kanchanathepsak et al.’s [20] RCT found no significant difference in the bloodless field between the recommended tourniquet pressure group (using LOP) and standard tourniquet pressure group. Drolet et al. [18] concluded that in an average sized patient, a tourniquet pressure of 200 mmHg would be sufficient to produce a bloodless field in most cases. When surgeons reported the adequacy of the surgical field [2,4] on a four-point scale, they were all reported as either excellent or good at lower tourniquet occlusion pressures. In Othman et al.’s study [16], where tourniquet pressures were gradually reduced, the incidence of breakthrough bleeding was 4.5% and was not found to be significantly associated with tourniquet pressures. Levy et al. [19] and Drolet et al. [18] also found the average tourniquet pressure to achieve a bloodless field to be below 250 (202 mmHg and 189.9 mmHg, respectively). All studies showed a bloodless surgical field at lower tourniquet occlusion pressures.

### 3.4. Secondary Outcomes

#### 3.4.1. Complications

The secondary outcomes reported include complications and procedure times. Sarfani et al. [3] reported that 13 out of 432 patients developed superficial wound dehiscence that healed by secondary intention, none of whom required a secondary procedure, and they thought this was unlikely related to tourniquet use. No complications were reported in the remaining studies where this was assessed. Kasem et al. [5] reported no evidence of tourniquet-induced pain immediately after the procedure, at 6 h, or the day after surgery.

#### 3.4.2. Operative Time and Tourniquet Time

Sarfani et al. found no difference in operative times between the groups with lower tourniquet occlusion pressures and in patients with the higher pressure of 250 mmHg. Kasem et al. [5] found no statistically significant difference in the tourniquet time when using the LOP versus the AOP to assess the lowest tourniquet pressure (*p* = 0.63). The tourniquet time ranged from 2 min [2] to 133 min [21] when reported.

#### 3.4.3. Cuff Width

Moore et al. [17] found that wider cuff widths were associated with lower inflation pressures required to eliminate blood flow.

##### Meta-Analysis

**Figure 1:**
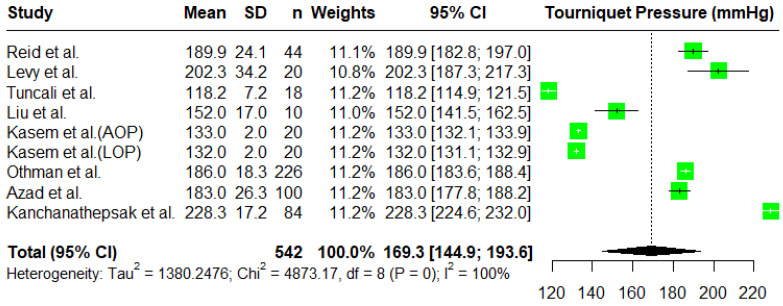
A forest plot of the meta-analysis for tourniquet pressures (mmHg) using a random effects model [2,4,5,7,16,19,20,21].

For a single-arm meta-analysis of the minimal tourniquet inflation pressures used to achieve a bloodless surgical field, eight research papers were included, and of those, two research papers reported outcomes in two different contexts. Reid et al. [7] reported the tourniquet inflation pressures for two groups, namely an upper extremities group and a lower extremities group. We excluded the results for the lower extremities group for this study. Kasem et al. [5] also reported two groups, where in one group, the tourniquet inflation pressure was estimated using the AOP, and in the other group, it was estimated using the LOP. Considering all reported results separately, we consider them as nine studies in our meta-analysis. In total, there were 542 patients treated with variable tourniquet inflation pressures. In some studies, there were groups of patients with a fixed tourniquet pressure, and they were excluded from this meta-analysis. The overall mean tourniquet inflation pressure was estimated using a random effects model to be 169.3 mmHg with a 95% C.I. given by (144.9, 193.6) (Figure 1). The largest pressure was reported by Kanchanathepsak et al. [20] with a mean tourniquet pressure of 228.3 mmHg. To compute the overall effect, we used a random effects model. However, the heterogeneity in the reported results is also significant (*p*-value < 0.0001). The I^2^ is 100%, which also suggests that the results obtained in the studies are very heterogenous.

**Figure 2:**
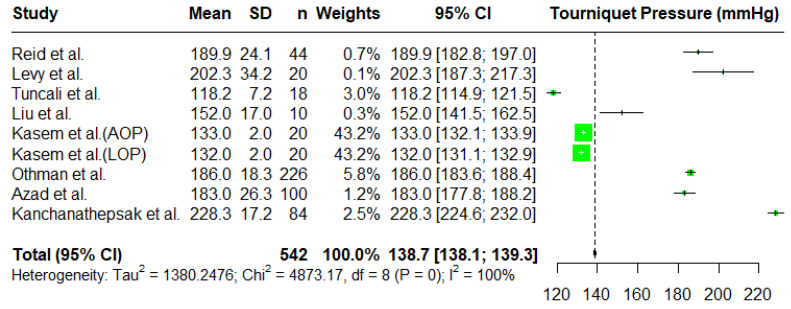
A forest plot of the meta-analysis for tourniquet pressures (mmHg) using a fixed effects model [2,4,5,7,16,19,20,21].

A sensitivity analysis was carried out to determine the effect of the statistical model used in the analysis. Figure 2 reports a forest plot from a fixed effects model with the overall mean estimated to be 138.7 mmHg with a 95% C.I. given by (138.1, 139.3). In a fixed effects model, most of the weights are given to the results in Kasem et al. [5] as they report very small standard deviations. The results are also very different from the random effects model. Due to large heterogeneity in the data, fixed effects models are not appropriate, and we observed that they produce estimates influenced solely by a smaller mean tourniquet inflation pressure.

**Figure 3:**
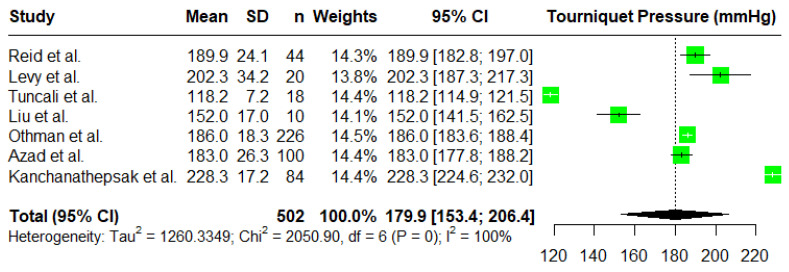
A forest plot of the meta-analysis for Tourniquet—Pressures—(mmHg) using a random effects model excluding the study by Kasem et al. [2,4,5,7,16,19,20,21].

As we observed that the reported standard deviations for Kasem et al.’s [5] study are quite low compared to other studies, we ran another sensitivity analysis without these two studies (AOP and LOP). The results of the random effects model without the inclusion of the study by Kasem et al. [5] are reported in Figure 3. The overall mean is estimated to be 179.9 mmHg with a 95% C.I. given by (153.4, 206.4). The results are not very different from those of the random effects model in Figure 1, and we observed that the random effects model is robust against the outlying studies with very small reported standard deviations.

**Figure 4:**
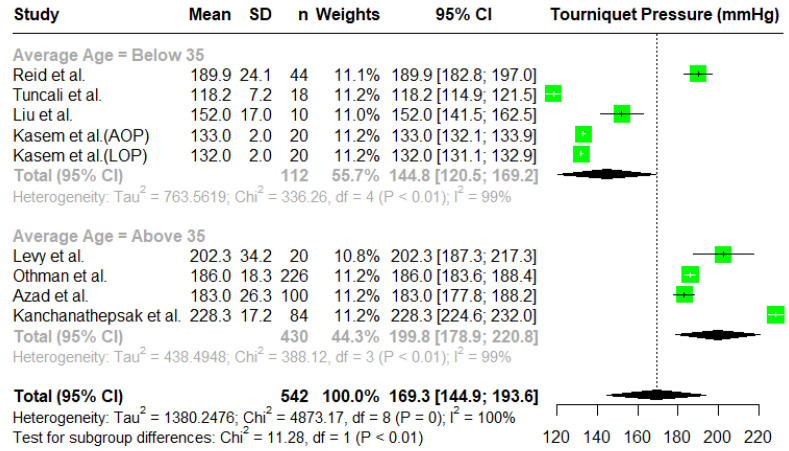
A forest plot of the subgroup analysis based on age [2,4,5,7,16,19,20,21].

To investigate the heterogeneity in the studies, we considered some subgroup analyses. All studies reported the ages of the participants, and we performed a subgroup analysis based on the average age of the patients. We divided the studies into two subgroups, namely a group with an average age below 35 and a group with an average age above 35. The results are reported in Figure 4. The overall estimated mean tourniquet inflation pressure for the subgroup of individuals below 35 years of average age is 144.8 mmHg (95% C.I. (120.5, 169.2)), and the overall estimated mean tourniquet inflation pressure for the subgroup of individuals above 35 years average age is 199.3 mmHg with a 95% C.I. (178.9, 220.8). There is a significant difference between the subgroups (χ^2^(1) = 11.28; *p* = 0.008).

**Figure 5:**
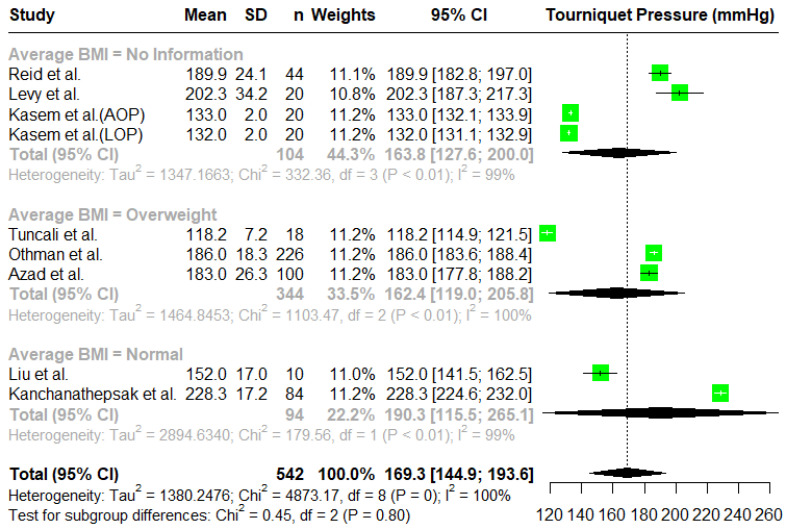
A forest plot of the subgroup analysis based on BMI [2,4,5,7,16,19,20,21].

We also performed a subgroup analysis based on body mass index (BMI). However, not all studies reported the BMI of the patients under study; hence, we created three subgroups, namely a group with no information on BMI, a group with a normal BMI, and a group with an overweight BMI. The results are presented in Figure 5. There are no significant differences between the subgroups based on BMI (*χ*^2^(2) = 0.045, *p* = 0.801).

The subgroup analysis showed that there are some heterogeneities due to the ages of the patients but not due to the BMI values reported. However, not all heterogeneity is explained by the ages of the participants as the heterogeneity within the subgroup of ages is also quite high. There may be other factors, like the procedures used, the baseline blood pressures, or the comorbidities of the patients, which can contribute to heterogeneity. Due to the lack of information on these factors reported in the studies, we could not carry out other sensitivity analyses or subgroup analyses to determine the source of the heterogeneity.

## 4. Discussion

The pneumatic tourniquet continues to remain an integral part of upper limb surgery to provide a bloodless field and allow for adequate visualization of anatomy by the surgeon. Although it was found that the conventional pressure of 250 mmHg allows for this, this systematic review and meta-analysis suggests that this value is unnecessarily high in many cases. This study suggests that it can be adjusted according to patient factors, such as limb size, BP, and comorbidities (as to whether the patient is able to tolerate a lower BP at anesthetic induction), as well as equipment factors such as the cuff width and modern pneumatic tourniquets, which allow for real-time monitoring of pressure and limb perfusion [22].

Our study suggests that a mean tourniquet pressure of 138.7 mmHg is comparable to the standard 250 mmHg value used in upper limb surgery in its ability to provide a bloodless surgical field and regarding associated complications such as pain. Some studies suggest taking into account the sBP [2,3,18,22], and others suggest using the AOP and LOP [4,5,20,21] in order to ascertain the minimal inflation pressure in upper limb surgery. The difficulty with regard to assessing the lowest effective tourniquet pressure intraoperatively using the LOP and AOP is that it requires additional time and calculation, which may translate to operating room inefficiency. It may also require operator skills for the determination of the occlusion pressure and potential investment in new materials, including a tourniquet which is able to measure the arterial pressure beneath the tourniquet [3]. Tuncali et al. [4] recommended a formula to calculate the AOP that must be applied in the operative setting related to the sBP of the patient divided by a predetermined tissue padding coefficient (K_TP_) in a patient with a limb circumference between 20 and 75 cm: [AOP = (sBP + 10)/K_TP_]. This may provide an alternative to methods which require a Doppler and other alternative equipment. Kasem et al. [5] measured the impact of hypotensive anesthesia on the minimal tourniquet inflation pressure and measured the time it takes to calculate the AOP (using the formula AOP = [sBP + 10]/K_TP_) and adding a safety margin of 20 mmHg versus LOP (using a Doppler ultrasound and then adding a 40 mmHg safety margin) intraoperatively in seconds and found that it was statistically faster to assess the AOP to adjust tourniquet pressure versus the LOP. However, when using both of these methods to assess the lowest effective tourniquet pressure, it was found that both groups provided an adequately bloodless surgical field.

The use of the intraoperative sBP may provide a more pragmatic approach to adjusting the pneumatic tourniquet pressure as it is routinely monitored intraoperatively, and it has consistently provided a bloodless surgical field in elective hand surgery [2]. Azad et al.’s study even found that when using lower tourniquet pressures, there was not a requirement to adjust or reinflate the tourniquet intraoperatively, and this study used pre-determined categories for inflation pressure based on the sBP (as shown in Table 3) [2]. Ultimately, these methods of calculating the minimum tourniquet pressure are influenced by theatre team preference and resources but highlight the need to move away from a pre-set value of 250 mmHg and to instead adjust the pressure according to patient factors. The use of hypotensive anesthesia for patients that can tolerate it should also be considered by the operating team to reduce the tourniquet pressure as it has shown to allow for lower tourniquet pressures.

The aforementioned joint BOA guidelines recommend a tourniquet pressure of systolic BP + 50–100 mmHg in the upper limb with reference to Tuncali et al.’s and Kasem et al.’s findings [4,5,6]. This suggests that in the majority of normotensive patients, the lowest effective tourniquet pressure lies below the fixed value of 250 mmHg. It also accounts for patient-specific sBP rather than set categories, as carried out in Azad et al.’s study [2].

An adequate surgical field is integral to upper limb surgery and, as mentioned, all the studies included in this review found that lower pressures provided an adequate view, which was further confirmed by the surgeon’s assessment of this view using a scale. Surgeons’ perceptions of the adequacy of the field remain an important part of assessment as it is their view of the operative field which will impact the tourniquet time and overall procedure time. However, one could argue that this remains a subjective measure and is dependent on factors such as surgeon experience. Robust objective methods are further required in order to assess the lowest effective tourniquet pressure.

When assessing further factors which can influence lower tourniquet inflation pressures, Moore et al. [17] measured pneumatic tourniquets with a width ranging from 4.5 to 15 cm and found that wider cuffs required lower tourniquet pressures. However, given the small sample size of 10, further work regarding the optimal cuff width in relation to tourniquet pressures is required to ascertain the optimal relationship. With regard to the tourniquet width, pressure, and pain, one study also reported that wider cuffs (14 cm versus 7 cm) at lower occlusive pressures result in less pain for patients, and wider cuffs are more effective at lower pressures. Conversely, using wider cuffs at high pressures caused more pain to patients and therefore highlights the balance required when using pneumatic tourniquet compression. This study also considered the tourniquet occlusion time and post-tourniquet pain and found that this influenced post-operative ischemic pain in patients [23,24].

Tourniquet-related complications are well documented in the literature. Although minimal complications were reported in the studies reviewed in this study, pain remains an important consideration. Surgeons are increasingly utilizing nerve blocks, namely intercostobrachial nerve blocks, to reduce postoperative pain associated with tourniquet use [25]. This can help minimize the pain resulting from prolonged tourniquet use. However, similarly to what was mentioned before, a further study identified that despite there being some benefit, pain is significantly related to tourniquet time, with one study suggesting that there is no statistically significant difference with regard to nerve blocks and post-operative pain post pneumatic tourniquet use [26]. It is important to consider that the patient is awake in many elective hand surgeries. Ensuring adequate tourniquet pressure to ensure an adequate visual field may also influence the procedure time.

### Limitations

There are several limitations in the evidence of using lower tourniquet pressures compared to the standardized pressure of 250 mmHg. The overall quality of evidence for outcomes are low due to the majority of the included studies being non-randomized studies. This is further undermined by the high risk of bias in each study as most studies relied on a subjective assessment of the bloodless field and did not consider controlling for cofounding factors. The scarcity of comprehensive outcome metrics presents a significant barrier to critically appraising the optimal tourniquet pressure, and the wide range of differences in tourniquet pressure applied and methods in determining the optimal pressure also compound this. The sample sizes in a few studies were small, which would have contributed to the random variation in the results.

The studies are very heterogenous owing to the differences in methods for assessing the lowest effective tourniquet pressure and outcomes. The studies included in the meta-analysis use a variety of parameters, including the LOP, AOP, and pre-determined equations and pressures determined by the sBP. Measurements of the adequacy of the surgical field varied from the surgeon’s perception during the operation, to a measure of the requirement to adjust the tourniquet intraoperatively, and to a measure of when capillary bleeding ceased. This is a limitation of this meta-analysis.

## 5. Conclusions

This systematic review highlights the benefit of the use of lower tourniquet pressures to produce an adequate surgical field and influence procedural outcomes. The current evidence suggests that there is no additional benefit of using the standardized tourniquet pressure of 250 mmHg. However, the findings of this systematic review and meta-analysis are limited by the quality of the individual studies. Given the notable risk of bias and heterogeneity, there is a requirement to conduct robust randomized controlled trials. We recommend that clinicians assess the sBP and use an appropriate tourniquet pressure accordingly without using the standardized value of 250 mmHg.

## Figures and Tables

**Table 1:** Risk of bias of studies included in this review using ROBINS-I tool [13,14].

Studies Assessed Using ROBINS-I Tool	Domain 1: Bias Due to Confounding	Domain 2: Bias Due to Selection of Participants	Domain 3: Bias in Classification of Interventions	Domain 4: Bias Due to Deviation from Intended Intervention	Domain 5: Bias Due to Missing Data	Domain 6: Bias in Outcome Measures	Domain 7: Bias in Selection of Reported Result	Overall Risk of Bias
Reid et al. [7]	no information	no information	moderate	low	low	serious	moderate	serious
Van Roekel and Thurston [15]	serious	no information	low	low	low	serious	moderate	serious
Othman et al. [16]	serious	low	moderate	low	low	serious	low	serious
Moore, Garfin, and Hargens [17]	serious	moderate	low	low	low	serious	moderate	serious
Drolet et al. [18]	serious	moderate	low	low	low	serious	low	serious
Levy et al. [19]	moderate	moderate	low	low	low	serious	low	serious
Sarfani et al. [3]	serious	low	low	low	low	serious	low	serious
Azad et al. [2]	serious	moderate	low	low	low	serious	low	serious

**Table 2:** Risk of bias for studies included in this review using ROB-2 tool [13,14].

Studies Assessed Using ROB-2 Tool	Domain 1: Bias Arising from Randomization Process	Domain 2: Bias Due to Deviations from Intended Interventions	Domain 3: Bias Due to Missing Outcome Data	Domain 4: Risk of Bias in Measurement of Outcome	Domain 5: Risk of Bias in Selection of Reported Risk	Overall Risk of Bias
Kasem et al. [5]	low	low	low	high	low	high
Tuncali et al. [4]	low	low	low	high	low	high
Kanchanathepsak et al. [20]	low	low	low	high	low	high
Liu et al. [21]	no information	low	low	high	low	high

**Table 3:** Summary of studies included in review and outcomes of lowest tourniquet occlusion pressure [2,3,4,5,7,15,16,17,18,19,20,21].

Author	Number of Participants Included in Review	Mean Age of Participants (Years)	Method for Determining Tourniquet Occlusion Pressure	Lowest Tourniquet Occlusion Pressure to Achieve Bloodless Field
Reid et al., 1983 [7]	44	Not stated	Doppler occlusion pressure + 50 mmHg and then adjusting accordingly	Mean: 189.9 mmHg Maximum: 225 mmHg
Van Roekel and Thurston, 1985 [15]	15	36.9	Commencing at 300 mmHg and then gradually decreasing pressure until capillary bleeding occurs	Maximum: 150 mmHg
Moore, Garfin, Hargens, 1987 [17]	10	Not stated	Elimination of Doppler flow	Range: 90 mmHg–160 mmHg
Levy et al., 1993 [19]	50	39	Based on mean BP: 1.68 × mean BP + 50	Mean: 202.3 ± 34.2 mmHg
Liu et al., 2013 [21]	10	31	AOP = 17.9 + 3.158 (upper extremity circumference (cm)) + 0.408 (systolic BP (mmHg))	Mean: 152.3 ± 16.7 mmHg
Drolet et al., 2014 [18]	505	40.1	Systolic BP of 250 mmHg, 225 mmHg and surgeon preference	Mean: 152.7 mmHg Maximum: 275 mmHg
Sarfani et al., 2016 [3]	228	59	Based on systolic BP—value of 125 mmHg, 150 mmHg, 175 mmHg, 200 mmHg, or 250 mmHg used	Range: 125 mmHg–250 mmHg
Kasem et al., 2020 [5]	40	26.4	AOP and LOP	Mean: 132 ± 2 mmHg
Othman et al., 2021 [16]	226	57	LOP	Mean: 187 mmHg
Tuncali et al., 2021 [4]	115	56.9	AOP + 20 mmHg and then elimination of Doppler flow	Mean: 175.5 ± 13.2 mmHg
Azad et al., 2022 [2]	100	50	Based on systolic BP—60 mmHg was added for BP < 130 mmHg, 80 mmHg for 131 and 190 mmHg, and 100 mmHg for >191 mmHg	Mean: 183 ± 26 mm Hg
Kanchanathepsak et al., 2023 [20]	84	56	LOP whilst monitoring distal arterial pulse	Mean: 228 ± 17.2 mmHg

**Figure A1:**
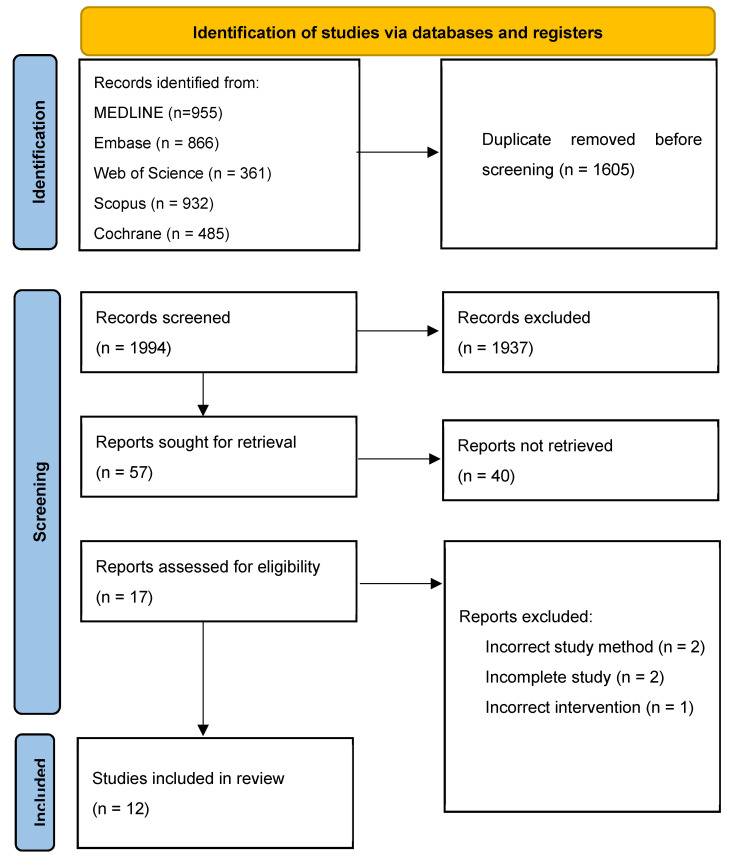
PRISMA Diagram for Identification of Studies Included in This Review [2,3,4,5,7,15,16,17,18,19,20,21] and exluded reports [27,28,29,30,31].

## Data Availability

All the papers included in this review are referenced.

## Supplementary Materials

The following supporting information can be downloaded at: https://www.mdpi.com/article/10.3390/jcm14061938/s1, PRISMA 2020 Checklist.
